# A giant floating thrombus in the ascending aorta: a case report

**DOI:** 10.1186/s12893-020-00983-6

**Published:** 2020-12-09

**Authors:** Peng Yang, Ya Li, Yao Huang, Chen Lu, Weitao Liang, Jia Hu

**Affiliations:** 1 Department of Cardiovascular Surgery, West China Hospital of Sichuan University, No. 37 Guo Xue Alley, Chengdu, 610041 China; 2 West China Clinical Medicine School, Si Chuan University, No. 17 Section 3, South Renmin Road, Wuhou District, Chengdu, 610041 China

**Keywords:** Floating thrombus, Ascending aorta, Surgery

## Abstract

**Background:**

A floating thrombus in an ascending aorta with normal morphology is very rare, but when it does occur, it may induce a systemic embolism or fatal stroke. The pathophysiological mechanisms of aortic mural thrombi remain unclear, and there is no consensus regarding therapeutic recommendations.

**Case presentation:**

We report a 49-year-old male who presented with chest discomfort for 5 days and was admitted to our emergency unit. A contrast-enhanced computed tomography angiography (CTA) surprisingly demonstrated a large filling defect suggestive of a thrombus in his otherwise healthy distal ascending aorta. Surgical resection of the mass and attachment site was performed. Histological examination confirmed that the mass was a thrombus, but the cause of the thrombus formation was unknown.

**Conclusions:**

floating aortic thrombi are rare, and they are prone to break off, thus carrying a potential risk for embolic events with catastrophic consequences. Surgical resection, both of the aortic thrombus and attachment site, as well as postoperative anticoagulant administration, are standard treatments.

## Background

An aortic mural thrombus (AMT) without an aneurysm or dissection is rare, with an incidence rate of about 0.45% [1]. It is occasionally identified incidentally, either by a source of systemic emboli or by computed tomography angiography (CTA). The most common locations for AMTs are the aortic isthmus, descending thoracic aorta and lower abdominal aorta, with the rarest location being the ascending aorta [2–4]. The pathogenesis and treatment strategies for AMTs are still limited to those found in case reports, and there is no consensus. We report on a 49-year-old male who presented with chest discomfort for 5 days and was admitted to our emergency unit. Contrast-enhanced computed tomography angiography (CTA) showed the presence of an ascending aorta active occupancy, which was successfully resected surgically. Histological examination of the mass confirmed that it was a thrombus.

## Case presentation

**Fig. 1:**
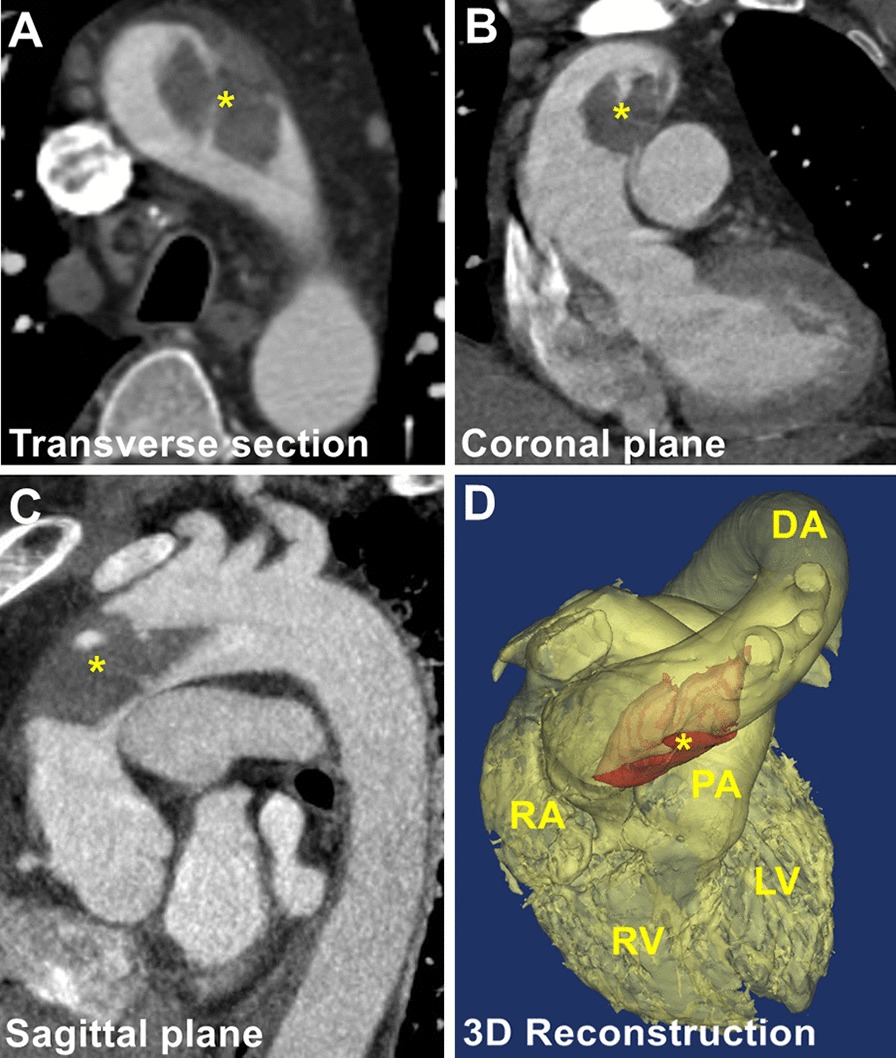
Preoperative CTA. Preoperative CTA shows a filling defect in the ascending aorta (yellow start). **a** transverse section; **b** coronal plane; **c** sagittal plane; **d** 3-D reconstruction. *LV* left ventricle, *RV* right ventricle, *RA* right atrium, *DA* descending aorta, *PA* pulmonary artery

A 49-year-old man presented with chest discomfort for 5 days and was admitted to our emergency unit. His medical history was unremarkable, except for cigarette smoking and a lower left limb embolic event that was treated by surgical embolectomy 3 years prior. Electrocardiography and laboratory tests were normal. A contrast-enhanced CTA surprisingly demonstrated a large filling defect suggestive of a thrombus in a healthy distal ascending aorta (Fig. 1, Additional file 1: Video 1). Further investigations, including autoimmune, thrombophilia, and blood culture studies were negative (Table 1), and so did his family.

**Table 1:** Hematologic test results

Parameters	Result	Parameters	Result
Prothrombin time	11.1 s	Red blood cell	5.74 × 10^12^/L
International normalized ratio	0.90	Hemoglobin	189 g/L
Activated partial thromboplastin time	27.6 s	White blood cell	6.23 × 10^9^/L
Thrombin time	18.8 s	Percentage of neutrophil granulocyte	69.4%
Fibrinogen	3.04 g/L	Platelet	182 × 10^9^/L
Alanine aminotransferase	16 IU/L	Alpha fetoprotein	3.10 ng/mL
Aspartate aminotransferase	18 IU/L	Carcinoembryonic antigen	2.11 ng/mL
Creatinine	59.0 μmol/L	CA15-3	13.29 U/mL
Estimated glomerular filtration rate	113.01 mL/min/1.73m^2^	CA19-9	1.02 U/mL
Triglyceride	2.97 mmol/L	CA-125	8.36 U/mL
Cholesterol	4.89 mmol/L	CA72-4	< 0.20 U/mL
High density lipoprotein	0.75 mmol/L	Neuron specific enolase	10.97 ng/mL
Low density lipoprotein	3.16 mmol/L	C-reactive protein	19.00 mg/L
Urea	2.30 mmol/L	Erythrocyte sedimentation rate	12.0 mm/h
Antinuclear antibody	Negative	Complement C3	1.0100 g/L
Anti dsDNA antibody	Negative	Complement C4	0.2730 g/L
Anti SM antibody	Negative	Rheumatoid factor	< 20.00 IU/mL
Anti SSA antibody	Negative	Properdin factor B	373.00 mg/L
Anti RNP antibody	Negative	Immunoglobulin G	10.60 g/L
Anti SSB antibody	Negative	Immunoglobulin A	3410.00 mg/L
Anti ScL-70 antibody	Negative	Immunoglobulin M	761.00 mg/L
CD3	81.8%	Immunoglobulin E	49.30 IU/mL
CD4	53.70%	Brain natriuretic peptide	22 pg/mL
CD8	24.30%	Troponin-T	16.1 ng/L

**Fig. 2:**
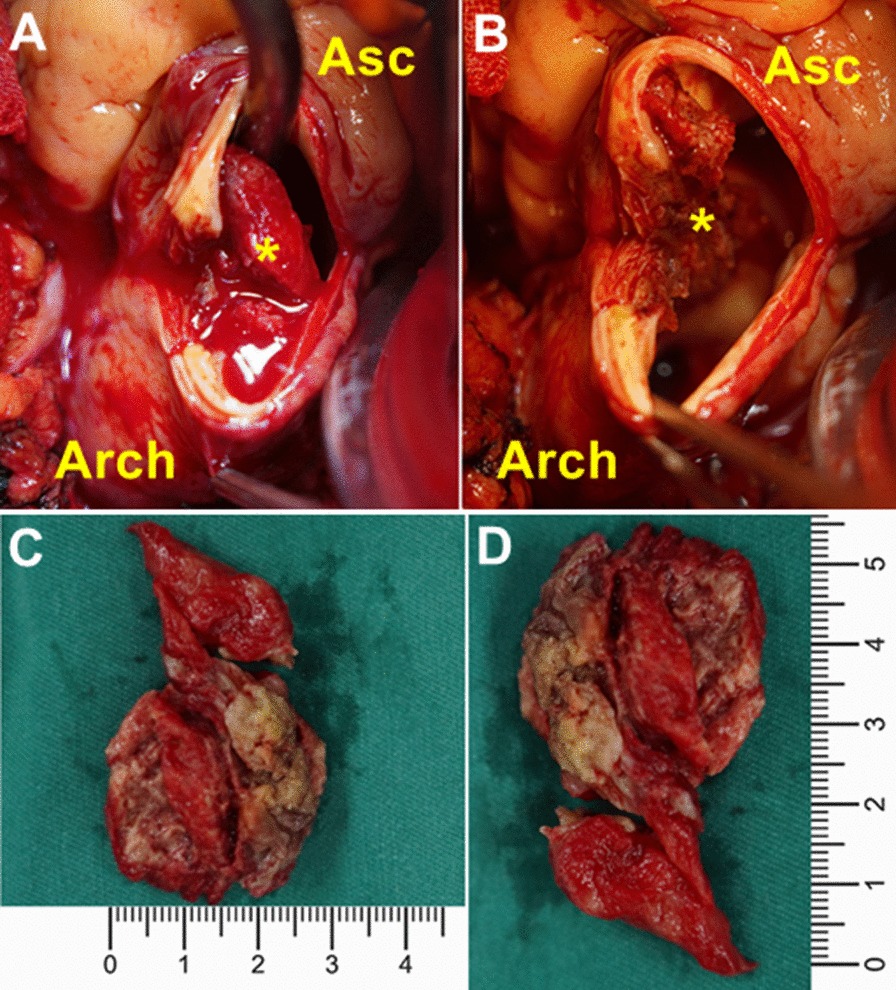
Intraoperative findings. **a**, **b** Floating ascending aortic thrombus. The aortic wall attachment site was unusually thick and fragile; **c**, **d** the size of the thrombus is 5.5 × 3.0 cm

For a young male with such a large floating thrombus in the ascending aorta, we chose surgical removal of the intra-luminal mass to avoid the recurrence of a peripheral or visceral embolism. After a discussion with professionals from multiple disciplines, we performed surgical intervention for this patient. Under general anesthesia, a preoperative transesophageal echocardiography showed a mobile lesion on the anterior wall of the distal ascending aorta (Additional file 2: Video 2). The surgery was performed through a standard median sternotomy on cardiopulmonary bypass after heparinized. The right femoral artery and the superior and inferior vena cavae were cannulated, with the intent of obtaining deep hypothermic circulatory arrest (at 24 ℃). The heart was arrested with retrograde cardioplegia, followed by retrograde cerebral perfusion and circulatory arrest. A transverse aortotomy was performed, and the ascending aorta and arch were carefully inspected. A 5.5 × 3.0 cm mixed thrombus was attached to the aortic wall at the junction of the ascending aorta and proximal arch (Fig. 2). After thrombus removal, an extensive area of intimal defect and an abnormally thick and fragile aortic wall was observed at the attachment site. To seal the prothrombotic area and avoid recurrence, the ascending aorta and proximal arch were replaced with a 28 mm Dacron vascular prosthesis conduit (Gelweave, Vascutek, Terumo, Inchinnan, UK). Histological examination of the aortic specimen confirmed the thrombotic nature of the structure, and no sign of connective tissue disorders or malignancy was detected. The postoperative course was uneventful, and the patient was discharged from the hospital 7 days after surgery. No complications were reported in 3-month follow-up. As we resected the aortic thrombus and its attachment stie, and the replaced artificial blood vessel had anticoagulated properties, no anticoagulation treatment was required for this patient. The CTA scan at the 3-month follow-up confirmed the stability of the grafts without a recurrence of an aortic thrombus.

## Discussion and conclusions

To the best of our knowledge, such a large floating thrombus in the ascending aorta that has not caused devastating complications has rarely been reported. The pathophysiological mechanisms of AMT remain unclear, although coagulopathy, immunological disorders, malignancies, intra-aortic atheroma, aortic structural abnormalities, trauma, steroid use, and substance abuse have all been suggested as possible causes [5, 6]. This was not the case for our patient, however.

Because of the rarity of the disease and the fact that many patients are asymptomatic before significant embolic events occur [7], early diagnosis of a floating aortic thrombus is difficult, and there is no accurate incidence rate of AMTs. In a study of 10,671 consecutive autopsies, however, the incidence of non-aneurysmal AMT was found to be about 0.45%, higher in women than in men [4].In this patient, who was admitted to our emergency unit for chest pain. The emergency ECG and laboratory test eliminated acute myocardial ischemia. CTA showed no aneurysm or aortic dissection, except for a floating thrombus in the ascending aorta. It's not clear whether chest pain has a causal relationship with the aortic mural thrombus, but the floating aortic thrombus does need further treatment.

AMTs are prone to break off, thus carrying a potential risk of cerebral, peripheral, or visceral embolic events with catastrophic consequences [8, 9]. The most common embolic site is the lower extremity artery, with the next most common sites being the mesenteric and renal arteries. The rarest embolic sites are the cerebral and coronary arteries, but these were often the most lethal and seriously affected the prognosis [9, 10]. Toyama et al. believed that the risk of thromboembolism is related to the size of the aortic thrombus base and the degree of calcification, but not to the location of attachment or volume of the thrombus [11]. The risk of peripheral or visceral embolism is 12% in a sessile thrombus, while it is 73% in a floating thrombus [4]. Approximately 6% of AMT may be the direct cause of death [1]. To describe the hemodynamic features and evaluate the break-off potential of lesions, Ruggero De Paulis et al. have defined a new parameter called the break-off risk ratio (boRR) [12]. This parameter refers to the length ratio of the floating and attached portions of the lesion, and a higher value indicates a higher possibility of the lesion breaking off. This may help in the selection of management strategies. However, further studies are required to verify this parameter’s significance.

A diagnosis of AMT mainly depends on imaging examinations. CTAs are conducive to locating aortic thrombi and judge whether there is an asymptomatic peripheral or visceral embolism, as well as its location [13], but the contrast agent and radiation are harmful. Transthoracic or transesophageal echocardiographies also have high accuracy rates and can evaluate the size, shape, attachment position, and aortic wall characteristics of thrombi located in the proximal ascending aorta [6]. However, a thrombus in the distal part of the ascending aorta and/or aortic arch cannot be accurately evaluated by echocardiography, due to the interference of gas in the trachea. In addition, magnetic resonance imaging has been reported to aid in the diagnosis of aortic thrombi and can help to exclude the possibility of malignancy [5]. This patient had a past medical history of acute right lower extremity embolism three years ago, which may come from the floating aortic thrombus. However, as limited by less clinical experience or some other reasons, the local hospital which performed previous surgical leg embolectomy didn’t do aorta imaging. Therefore, comprehensive and systematic preoperative examinations are very important to identify the source of embolus. In general, CTA is a first-line examination because of its convenience and high sensitivity [13]. Moreover, our case suggests that CTA, followed by transesophageal echocardiography, can provide optimal visualization of the AMT for accurate diagnosis and risk evaluation, and it is helpful in determining safer surgical procedures and the extent of resection.

With regard to treatment strategies, there is no consensus on therapeutic recommendations. A review of the literature from the past 5 years indicates that a conservative approach with anticoagulants or endovascular or open surgical interventions were all reasonable treatment options for floating aortic thrombi (Table 2), depending on their size, location, mobility, and any related peripheral embolisms. Conservative treatment is preferred and considered to be the cornerstone of successful treatment by many researchers [2, 26]. Thrombolytic drugs, like tissue plasminogen activator, can rapidly break up the thrombus, but they increase the risk of embolism as the thin attachment site, instead of the thrombus itself, may be lysed first [18]. In contrast, anticoagulants, such as warfarin and rivaroxaban, are much safer for use. Once the aortic thrombus is diagnosed, anticoagulation treatment should be started as early as possible [2]. However, some researchers have expressed concern that as many as 25% of patients treated with anticoagulants alone ultimately need surgical treatment due to continuous or recurrent thrombi [2, 29]. Additionally, the appropriate drug, dose, and treatment duration are all important factors in the conservative treatment of thrombi, but consistent guidelines for their use have not yet been established [4]. Therefore, conservative treatment is more likely to be performed in asymptomatic patients with small sessile thrombi or in those who are unable to bear surgery.

**Table 2:** Literature review

Author et al	Year	Age	Gender	Location	Symptom	Treatment
Schattner et al. [14]	2016	72	M	Proximal descending aorta	Multiple splenic infarcts	Surgery and anticoagulant
Pang et al. [15]	2016	49	M	Distal ascending aorta	Renal infarction	Surgery and anticoagulant
Keraliya et al. [16]	2016	22	M	Aortic root	Myocardial infarction	Anticoagulation therapy
Teranishi et al. [8]	2016	61	M	Ascending aorta	Gallbladder, superior mesenteric artery, and both renal arteries embolism	Surgery and anticoagulant
Siani et al. [17]	2016	62	F	Descending thoracic aorta	Bilateral lower limb embolism	Endovascular treatment and anticoagulant
Weiss et al. [18]	2016	71	F	Aortic arch	Left brachial artery embolism	Surgery and anticoagulant
	2016	51	F	Aortic arch	Infrarenal aorta embolism and paraplegia	Surgery and anticoagulant
	2016	59	F	Aortic arch	Superior mesenteric artery embolism	Surgery and anticoagulant
	2016	47	M	Aortic arch	Acute chest pain	Anticoagulant
	2016	48	F	Aortic arch	Left middle and anterior cerebral artery embolism	Surgery
	2016	77	F	Aortic arch	Left subclavian artery embolism	Anticoagulant
	2016	55	F	Aortic arch	Right femoral bifurcation embolism	Surgery and anticoagulant
	2016	57	F	Aortic arch	Left subclavian and brachial artery embolism	Surgery and anticoagulant
	2016	50	M	Aortic arch	Asymptomatic	Anticoagulant
	2016	82	F	Aortic arch	Subtotal occlusion of all supra-aortic vessels embolism	Endovascular treatment
Poon et al. [19]	2017	41	M	Ascending aorta	Lower abdominal pain	Surgery
Luetkens et al. [20]	2017	86	F	Ascending aorta	Dyspnea and severe sepsis	Unknown
Ozaki et al. [9]	2017	37	M	Ascending aorta	Acute myocardial infarction	Surgery and anticoagulant
Auer et al. [10]	2017	89	M	Ascending aorta	Chest pain and right sided hemianopsia	Anticoagulant
Maat et al. [21]	2017	48	M	Ascending aorta	Right renal embolism	Surgery and anticoagulant
Yang et al. [3]	2017	47	M	Ascending aorta	Superior mesenteric artery embolism	Surgery
	2017	62	M	Aortic isthmus	Splenic infarctions	Anticoagulant
	2017	73	M	Aortic isthmus	Chest pain	Anticoagulant
	2017	18	M	Thoracic aortic stent	Asymptomatic	Anticoagulant
	2017	56	F	Junction of stent body and iliac limbs	Asymptomatic	Observation
Avelino et al. [22]	2017	32	F	Distal aortic arch	Liver, pancreas, left kidney and spleen infarctions	Surgery
Kandemirli et al. [23]	2018	48	F	Extending from the aortic arch into the superior mesenteric artery	Superior mesenteric artery embolism	Surgery
Toyama et al. [11]	2018	72	M	Ascending aorta	Pulmonary embolism	Anticoagulant
Tigkiropoulos et al. [24]	2018	52	F	Aortic arch	Left lower extremity embolism	Anticoagulant and surgery
Sosa et al. [25]	2018	68	M	Descending thoracic aorta	Splenic and renal infarctions	Anticoagulant
Campanile et al. [26]	2019	63	F	Ascending aorta	Myocardial infarction and left upper limb ischemic	Surgery
Wang et al. [13]	2019	56	M	Ascending aorta	Asymptomatic	Surgery and anticoagulant
Dalal et al. [27]	2020	73	F	Distal ascending aorta	Chest pain and left leg claudication	Surgery
Gueldich et al. [28]	2020	43	F	Distal ascending aorta	Left upper limb recurrent acute ischaemia	Surgery
	2020	63	F	Ascending aorta	Left upper limb acute ischaemia, splenic infarctions and embolism of the right renal artery	Surgery

Endovascular treatment has the advantages of being minimally invasive and having fewer complications, providing a new choice for patients for whom conservative drug treatments are ineffective. However, thrombus fragmentation may be caused by guidewire movement or stent compression, which is why there is still a high rate of new embolism formation in the perioperative period. Careful management of the guidewire, at least 2 cm of the proximal and distal anchorage area, oversized less than 5%, and reassessment of the mesenteric and lower extremity artery patency at the end of the procedure are essential [4, 5]. Furthermore, adjustable sheath assisted guidewires and intravascular ultrasound promoted precise positioning of stents may help to reduce the risk of thrombus fragmentation. Andrea Siani et al. also recommended to reduce the dose and injection speed of the contrast agent and to install an intravascular filter or balloon [17]. In brief, endovascular treatment may be effective for resolving AMTs, but the long-term effect is still unknown. Surgical excision of the thrombus and aortic attachment site is another choice that the clinician has, especially when embolic events have occurred. Extracorporeal circulation and circulatory arrest are required during the therapy, however. Therefore, it is of great importance to evaluate both the perioperative risks and benefits of aortic arch surgery. It is also crucial to distinguish the floating aortic thrombus from aortic arch atheroma or debris, both of which carries a much higher perioperative risk if surgically removed. One study reported the local recurrence of a thrombus at the same attachment site after thrombectomy [18], indicating that the resection of the attachment site should be taken into consideration. Surgical resection, both of the aortic thrombus and the attachment site, became necessary for our case because the floating thrombus was so large and presented such a high risk for future embolic events.

In conclusion, floating aortic thrombi are rare, and they are prone to break off, thus carrying a potential risk for embolic events with catastrophic consequences. The pathophysiological mechanisms of AMT remain unclear, and there is no consensus on therapeutic recommendations. For a suspected ascending aortic floating thrombus, we advocate CTA, combined with transesophageal echocardiography, for a comprehensive assessment of an AMT. Surgical resection, both of the aortic thrombus and attachment site, as well as postoperative anticoagulant administration, are standard treatments. However, elderly patients or those with an extremely high risk from surgery can choose conservative drug treatment or endovascular treatment, if necessary.

## Supplementary Information

**Additional file 1: Video 1.** Preoperative CTA 3D reconstruction.

**Additional file 2: Video 2.** The preoperative transesophageal echocardiography showed a mobile lesion on the anterior wall of the distal ascending aorta.

## Data Availability

Not applicable.

## Abbreviations

AMT: Aortic mural thrombus
CTA: Computed tomography angiography
Asc: Ascending aorta
LV: Left ventricle
RV: Right ventricle
RA: Right atrium
DA: Descending aorta
PA: Pulmonary artery
